# Grave Sequelæ of a Renal Calculus

**Published:** 1860-11

**Authors:** W. H. Triplett

**Affiliations:** Woodstock, Shenandoah County, Virginia


ART. V.—*Grave Sequelæ of a Renal Calculus.* By W. H. TRIPLETT, M.D., of Woodstock, Shenandoah County, Virginia.

ON the 1st of September, 1859, I was called to see Philip Sheets, a farmer, fifty years of age, who was attacked with hematuria and retraction of the left testicle, with considerable pain along the spermatic cord and in the left lumbar region, and a tickling sensation at the end of the urethra. His pulse was eighty, appetite unimpaired, and the functions of the skin were performed naturally. Supposing the presence of a calculus in the pelvis of the kidney, or, more likely, traversing the left ureter, I ordered him a mild aperient, followed by a Dover's powder at bedtime. Under this treatment, continued for several days, the suffering was much alleviated and the urine assumed its natural color, having an acid reaction and a specific gravity of 1·020.

In the course of four or five days symptoms of vesical irritation set in, indicating that the calculus had traversed the ureter and become lodged in the bladder. Constant and most urgent desire to urinate was a distressing symptom, and the act caused the patient great pain, especially when the flow of urine was interrupted by the calculus blocking up the orifice of the urethra. The testis at the same time was very large, hard, and painful, and there was an erysipelatous blush of the scrotum over the affected gland. The skin was dry and hot, the pulse eighty-three, and there was loss of appetite and sleep. A vigorous saline cathartic was administered, cold medicated lotions were applied to the scrotum, and blue mass was given in small doses at short intervals. Active treatment did not, however, yield its equivalent of benefit; for although the orchitis was checked, the organ still remained indurated and enlarged, and micturition was attended with pain. For the latter condition, an infusion of flax-seed and uva ursi was found to afford more relief than any other remedies.

On the tenth of September I found him complaining of pain in the region of the prostate gland, and, upon an examination, I detected a fullness of the perineum over the organ. On the previous evening he had had a rigor; his pulse was frequent and intermitting, and his tongue deeply furred, and I was apprehensive that these signs were denotive of prostatic abscess. Subsequent events confirmed these fears. Quinine and opium were now freely given, and in seventy-two hours the abscess burst into the rectum, forming a recto-vesical fistule, discharging large quantities of thick, yellow pus, but at no time did urine seem to escape from the anus. This secretion was highly charged with pus, or, more correctly speaking, there was scarcely any discharge of urine at all, the bulk of the discharge through the urethra being a mixture of pus and mucus. The escape of pus from the urethra and rectum clearly denoted the formation of a fistule, although no urine could be detected flowing from the latter outlet. During this stage of renal excitement, however, it is proper to state that not a pint of urine was secreted in a week, and that was highly ammoniacal, and remained so for some time subsequently. When the abscess had discharged its contents there was an amendment in all the symptoms. The pulse returned to its natural standard, the appetite improved, and the patient was refreshed by a sound sleep.

The history of this case beautifully illustrates the trite principle in pathology, that two severe inflammations cannot go on at the same time. When the symptoms of prostatic disease were strongly expressed, the inflammation of the testicle began to abate, and was reduced to a certain point, where it remained stationary until the maturation of the former affection and the bursting of the abscess, when it resumed its line of march, and, notwithstanding every effort, progressed through all its stages. The nodulated testicle softened at many points, and opened on its posterior surface, when in the course of a week it was nearly well.

What became of the calculus it is impossible to say, since the most careful exploration of the bladder with a sound, and repeated examinations of the discharges, failed to discover it. My own supposition is that it has either become sacculated, or dropped down into the vesical orifice of the fistule.

